# Mental Control in Musical Imagery: A Dual Component Model

**DOI:** 10.3389/fpsyg.2019.01904

**Published:** 2019-08-21

**Authors:** Katherine N. Cotter

**Affiliations:** Department of Psychology, University of North Carolina at Greensboro, Greensboro, NC, United States

**Keywords:** musical imagery, mental control, involuntary musical imagery, auditory imagery, conceptual analysis

## Abstract

Hearing music in your head is a ubiquitous experience, but the role mental control plays in these experiences has not been deeply addressed. In this conceptual analysis, a dual-component model of mental control in musical imagery experiences is developed and discussed. The first component, initiation, refers to whether the musical imagery experience began voluntarily or involuntarily. The second component, management, refers to instances of control that occur after the experience has begun (e.g., changing the song, stopping the experience). Given the complex nature of this inner experience, we propose a new model combining and integrating four literatures: lab-based auditory imagery research using musical stimuli; involuntary musical imagery; mental rehearsal and composition in musicians; and *in vivo* studies of musical imagery in everyday environments. These literatures support the contention that mental control of musical imagery is multi-faceted. Future research should investigate these two components of mental control and better integrate the diverse literatures on musical imagery.

## Introduction

Engagement with music is pervasive—we subscribe to music-listening services, star in mini-concerts during our showers and commutes, and are bombarded by upbeat, bopping tunes when shopping. Our musical experiences are not limited to our external environment, however—we also hear music in our “mind’s ear.” Musical imagery can broadly be described as hearing music in one’s head not simultaneously present in the environment (Bailes, 2007; Cotter et al., 2018). People report hearing musical imagery often in everyday life (approximately 25% of the time, Liikkanen, 2011; Cotter et al., 2018).

These common internal experiences also vary in their complexity. In some experiences, people only imagine select components of the music (e.g., melody, vocals) but in others report experiencing more subtle components of the music, such as harmonic lines or different timbres of instruments (Bailes, 2007). Further, these experiences need not be solely auditory. In many cases, people’s musical imagery experiences are multimodal and include visual or kinesthetic imagery (e.g., Bowes, 2009) or involve moving or humming to the imagined music (e.g., Cotter et al., 2018). Musical imagery can be embedded in rich internal narratives, such as envisioning yourself performing in a desired role (Bowes, 2009), or echo our current state of mind and personal concerns (Floridou et al., 2015). Musical imagery is a dynamic, complex phenomenon; however, here I focus on only the auditory components of these experiences.

One multi-dimensional model of musical imagery (Cotter et al., 2018) identifies five qualities of everyday musical imagery experiences—valence, repetitiveness, vividness, length, and mental control. Most research on musical imagery in daily life has emphasized describing the what of the experience—what song, what trigger, what valence, what length. Less attention has been given to how these experiences unfold, change, or stop. The dimension regarding the mental control of musical imagery—an intriguing process involved in starting, stopping, shaping, and maintaining musical imagery—is complex, nuanced, and relatively understudied. Mental control can be further broken down into two primary components—initiation and management (Cotter et al., 2018). Initiation refers to how the episode of musical imagery begins—was it started on purpose or did it appear spontaneously? Management refers to attempts to control the musical imagery episode after it has begun. Management can take different forms, and research has focused on a few flavors of management: altering components of the music (e.g., pitch, tempo), sheltering and sustaining the experience amid distractions, and stopping the experience (e.g., Holmes, 2005; Beaman and Williams, 2010; Williamson et al., 2014; Cotter and Silvia, 2018). When thinking about control this way, it becomes evident that one episode of musical imagery can be controlled in one way but involuntary in another.

By discussing mental control of musical imagery this way, we can reflect on what the field has already examined and where we have yet to go. When re-examining the research traditions in musical imagery, it’s evident this framework provides new ways of organizing and interpreting what we know about mental control of musical imagery and demonstrates that seemingly different musical imagery experiences have more in common than they first appear to. Additionally, by having a common language with which to describe these mental control processes, we can better articulate what we already know, develop research questions that become obvious once operating within this framework, and refine our assessment of mental control.

In this conceptual analysis, I focus on only the dimension of mental control and examine what is known about the mental control of musical imagery in psychology and musicology. I demonstrate that mental control of musical imagery can be broken down into two distinct components—initiation and management. I analyze lab-based auditory imagery work using musical stimuli and explore how the principles of mental control established through auditory imagery research can be applied to musical imagery experiences in daily life. Because musical imagery in the lab and in daily life are related experiences, these principles can be further refined to address the greater complexity inherent in musical imagery in everyday life. This conceptual analysis also applies the dual component model to three everyday musical imagery approaches: involuntary musical imagery, mental rehearsal and composition, and ecological musical imagery (which emphasizes assessing musical imagery as it is happening in people’s everyday lives).^1^

## Key Concepts From an Auditory Imagery Approach to Musical Imagery

Musical imagery is one example of auditory imagery. Auditory imagery research, rooted in cognitive psychology, has often used tonal and musical stimuli in its lab-based paradigms to investigate principles of people’s auditory imagery experiences—this section focuses only on studies using musical stimuli. These music-based auditory imagery studies assess a range of people’s auditory imagery capabilities, from simple imagery-assisted pitch discrimination to complex mental transformations of melodies. Although this literature does not formally discuss mental control, the natures of the tasks provide support for the two proposed components of mental control—initiation and management. Table 1 provides descriptions of the tasks used in auditory imagery research.

**TABLE 1:** Descriptions of auditory imagery tasks.

**Methodology**	**Description**	**Studies**
Pitch Discrimination	Participants are presented with auditory stimuli (e.g., tones, song excerpts) and imagine music related to the initial stimuli, such as replicating it or imagining the continuation of the excerpt. People’s images are then probed for pitch accuracy by determining whether a target tone or musical notation matches their imagery. These tasks often require people to sustain their images.	Janata and Paroo, 2006; Herholz et al., 2008; Bailes et al., 2012; Weir et al., 2015
Timing Judgment	Participants listen to the beginning of a song excerpt and imagine the continuation of the excerpt. People’s images are then probed for timing accuracy—participants are presented with music from the same excerpt and determine whether it is in time with their image or is appearing too early or late. These tasks often require people to sustain their images.	Bailes and Bigand, 2004; Janata and Paroo, 2006; Weir et al., 2015
Temporal Accuracy	Participants are instructed to imagine music excerpts of varying lengths. For each excerpt, participants indicate when they have imagined the full excerpt. These tasks often require people to sustain their images.	Halpern, 1988; Halpern and Zatorre, 1999
Lyric Comparison	People are shown lyrics from well-known songs with two of the lyrics capitalized (e.g., happy BIRTH-day to YOU). Participants then determine whether the second capitalized lyric is on a pitch higher or lower than the first capitalized lyric. These tasks often require people to sustain their images.	Zatorre and Halpern, 1993; Aleman et al., 2000
Loudness Profile	People listen to a musical excerpt that varies in loudness during the passage. Participants then imagine the same excerpt, including its loudness profile, and use a slider to indicate the loudness profile of their image. These tasks often require people to sustain their images.	Bailes et al., 2012; Bishop et al., 2013a
Contour Tracking	People hear short melodies. People imagine each melody and indicate whether the pitch of a note was higher, lower, or the same as the prior note. These tasks often require people to sustain their images.	Weber and Brown, 1986
Tempo Judgment	People listen to or imagine excerpts of well-known and familiar songs. People then indicate the tempo of the music by tapping with their finger to the beat or by using a dial to adjust the speed of a click track so it matches the beat of the music. These tasks often require people to sustain their images.	Jakubowski et al., 2015, 2016
Pitch Manipulation	Participants are presented with initial tone(s) and manipulate the pitch of the tones to be higher or lower as specified. People then complete a pitch discrimination task. These tasks require people to alter the pitches of their images.	Hubbard and Stoeckig, 1988; Gelding et al., 2015
Melody Transformation	Participants hear a melody and are presented with a test melody that has been transformed—in a new key or reversed—or an untransformed control melody. People indicate if the test melody, when transformed, matches the first melody. These tasks require people to alter the key or temporal order of an excerpt using imagery.	Foster et al., 2013

### Initiating Musical Imagery

Inherent in any auditory imagery task is the need to construct a mental image. In early work, the imagery tasks were relatively simple—imagining the pitch of a presented tone and completing a signal detection task (Farah and Smith, 1983). These results suggest that people can form images of single tones at will, and these images facilitate auditory perception via a reduced detection threshold for imagined pitches as compared to non-imagined pitches. In Pitch Discrimination tasks (see Table 1), participants imagine specified tones, chords, or short passages of music (e.g., musical scales, simple melodies) and assess whether auditory probes match the pitch of their constructed image (Janata and Paroo, 2006; Herholz et al., 2008). On average, people demonstrated the ability to form the requested images with reasonable accuracy for single tones and chords (60–95% correct; Hubbard and Stoeckig, 1988), musical scales (78% correct when probe in tune; Janata and Paroo, 2006), and simple melodies (60 and 87% correct for non-musicians and musicians, respectively; Herholz et al., 2008). Overall, this suggests people can initiate musical images when asked.

### Sustaining Musical Imagery

Several studies also assess people’s ability to manage their established images, such as deliberately sustaining the image—Hubbard’s (2018) recent review of auditory imagery suggests this may be an overlooked dimension of control. In several Pitch Discrimination and Timing Judgment studies (see Table 1), participants hear the first few notes of a musical passage and imagine the remainder of the passage to determine whether a subsequent probe tone matches the pitch or timing of the imagined music (Bailes and Bigand, 2004; Janata and Paroo, 2006; Herholz et al., 2008; Weir et al., 2015). In one Timing Judgment study, participants were instructed to imagine the continuation of music for as long as possible and, when they were no longer able to continue the imagined music, to “check in” with the actual progression of the song by raising the volume of the stimulus song (Bailes and Bigand, 2004). The results indicated that the check-ins were related to structural properties in the music, suggesting that people can sustain images of sections of music, but when the piece shifts to a new section, people have difficulties imagining these transitions.

Other sustention work uses Temporal Accuracy tasks (see Table 1), which ask participants to indicate when their image of the passage reached the end (Halpern and Zatorre, 1999) or when they reach a specific point in the excerpt (Halpern, 1988). In Lyric Comparison studies (see Table 1), people are presented with two lyrics from a well-known tune (e.g., “Happy Birthday”) and are asked which of two lyrics has an associated note higher in pitch (Zatorre and Halpern, 1993; Aleman et al., 2000). In these basic sustention studies, people are able to maintain short images of familiar tunes (Zatorre and Halpern, 1993; Aleman et al., 2000; Herholz et al., 2008; Weir et al., 2015) and musical scales (Janata and Paroo, 2006) to perform the Pitch Discrimination and Timing Judgment tasks, but they tend to have more accurate pitch discrimination than timing judgments (Janata and Paroo, 2006; Weir et al., 2015).

Researchers have also used more complicated sustention tasks that involve continuous monitoring of an image. A more complex Pitch Discrimination task involved listening to a melody and judging whether the subsequently presented notation matched the heard melody (Bailes et al., 2012). To evaluate similarity, participants needed to generate an image of the notation and monitor their image for deviations from the target melody—on average, participants made accurate judgments approximately 70% of the time. Additionally, Contour Tracking work (see Table 1) finds that people can monitor changes in pitch across a musical passage via reporting whether a pitch is higher or lower than the one that immediately preceded it (Weber and Brown, 1986).

In Loudness Profile studies (see Table 1), participants listened to passages of music, paying special attention to the loudness throughout the piece. They then imagined the musical passage and indicated the dynamic contour of the piece using a slider during both the listening and imagining portions (Bailes et al., 2012; Bishop et al., 2013a). People were able to produce a dynamics profile of the imagined passage similar to the dynamics profile produced when listening to the passage.

Other studies using Tempo Judgment paradigms (see Table 1) ask participants to listen to or imagine specific pieces of music and indicate what they believed to be the correct tempo (Jakubowski et al., 2015, 2016). Unsurprisingly, people are most accurate when listening to a song (Jakubowski et al., 2016). Interestingly, increased physiological arousal influences tempo judgments—people chose faster tempos for both perceived and imagined music after a physical versus mental task (Jakubowski et al., 2015). In both Tempo Judgment studies, however, people were able to sustain their image to complete the tasks. Collectively, this work demonstrates people’s ability to maintain a musical image and suggests that in addition to making single, isolated judgments about their musical imagery (i.e., pitch discrimination, timing accuracy) people can monitor temporal qualities of their musical imagery.

### Manipulating Musical Imagery

Although sustaining musical imagery is one example of management, the more intuitive sense of management is the ability to manipulate and alter aspects of an image. In one Pitch Manipulation study (see Table 1), participants were presented with a single tone or chord and asked to imagine the tone or chord one step higher—their image was then probed for accuracy (on average 60–95% correct; Hubbard and Stoeckig, 1988). In a more complex Pitch Manipulation study, participants were presented with the first few notes of ascending or descending scales and imagined subsequent notes that were higher or lower in pitch as specified via up or down arrows (Gelding et al., 2015). After imagining multiple notes, a probe tone was presented for a pitch discrimination judgment to assess the accuracy of participant’s images. Musicians tended to be more accurate than non-musicians (82 vs. 76% accuracy, respectively).

Researchers have also examined people’s ability to perform complex mental manipulations using a Melody Transformation task (see Table 1; Foster and Zatorre, 2010; Foster et al., 2013). Musicians were presented with a target melody and needed to determine whether the test melody was the same as or different from the target. The test melody, however, was heard in one of three forms: reversed melodies (i.e., the melody was played from the end to the beginning), transposed melodies (i.e., the melody was played in a different key), and control melodies (i.e., the melody had not been transformed). To determine whether the test and target melodies were identical, participants needed to mentally transform the test melody to be in the same key or temporal order for comparison. Unsurprisingly, people were most accurate when presented with control melodies (between 76% and near 100% accuracy) and were less accurate when presented with transposed (69–90% accuracy) and reversed melodies (80% accuracy; Foster and Zatorre, 2010; Foster et al., 2013). These findings suggest that manipulations people make to their musical imagery can vary in complexity and difficulty.

Additionally, a survey measure—the Bucknell Auditory Imagery Scale (BAIS; Halpern, 2015)—has been used to assess the vividness and control of auditory imagery. In the vividness subscale, people are instructed to generate images of specific auditory experiences (e.g., a trumpet playing the beginning of “Happy Birthday”) and rate the lifelikeness of the resulting image. To assess control, people perform changes to the established auditory images (e.g., the trumpet stops playing and a violin finishes the song), like the management component of the proposed model, and indicate the ease of performing these manipulations. Not all items involve musical imagery, however—several items involve manipulating auditory images of environmental sounds (e.g., waves crashing against rocks on a beach) or human voices (e.g., the sound of an elderly clerk assisting you over the phone).

Performance on the BAIS-Control subscale suggests there are individual differences in the self-reported ability to control auditory images and, in some cases, predicts performance on auditory imagery tasks. In several cases, higher self-reported imagery control predicted better accuracy (Gelding et al., 2015; Greenspon et al., 2017) on auditory imagery tasks involving pitch judgments, and people who are more accurate in singing specific pitches (Greenspon et al., 2017) or have more musical experience (Gelding et al., 2015) report better control abilities. Interestingly, people reporting better control abilities were better able to predict changing beat intervals (as opposed to reacting to changes in beat intervals) during a sensorimotor synchronization task requiring updating beat representations while listening to music (Colley et al., 2018). People with higher BAIS-Control subscale scores were also better able to synchronize with the music (Colley et al., 2018). In other cases, however, the BAIS-Control subscale was unrelated to tempo-related judgments, such as tapping to the beat of imagined or heard music (Jakubowski et al., 2016).

### Limitations of the Auditory Imagery Approach to Musical Imagery

This work, however, has not explored the complexity of these experiences outside of the lab. In classic auditory imagery studies, the stimuli are single tones or chords or simple melodic lines (e.g., Farah and Smith, 1983; Hubbard and Stoeckig, 1988; Janata and Paroo, 2006) and more recent studies have used both simple (e.g., Foster et al., 2013; Gelding et al., 2015) and somewhat more complex stimuli (e.g., Bailes et al., 2012; Weir et al., 2015). But the considerable heterogeneity and idiosyncratic nature of everyday musical imagery contents has not been captured in the lab-based auditory literature. Often, everyday musical imagery contains familiar, recently heard songs (Liikkanen, 2008, 2011; Williamson and Jilka, 2014); other times, people use musical imagery as a tool to develop original compositions (Cowell, 1926; Mountain, 2001) and to rehearse for performances (Holmes, 2005; Bailes, 2007). Thus, a focus on only musical imagery experiences in controlled lab settings paints an incomplete picture of people’s musical imagery experiences outside of the lab. Indeed, the mind wandering literature has documented both similarities and differences between lab and daily life mind wandering experiences (McVay et al., 2009; Kane et al., 2017).

When applying this mental control framework to daily life, it is necessary to recognize that initiation and management of musical imagery in daily life will not look identical to these components when examined in a lab. First, in lab studies participants initiate and manipulate musical imagery as specified by researchers. Although possible, it is unlikely that in daily life someone is playing the first few notes of the Bb major scale and requests that another person imagines the rest of that scale or for someone to be told to imagine a single tone and be asked, “Is this your note?” People have a variety of motives for deliberately initiating and managing musical imagery that are not captured in lab experiments. Additionally, the nature of lab-based auditory imagery tasks is predicated on people willfully initiating and managing specific musical images, making it difficult to study involuntary or uncontrolled instances of musical imagery (see Hubbard, 2018); however, recent studies have begun to induce involuntarily initiated musical imagery experiences to better understand these processes (e.g., Hyman et al., 2013; Floridou et al., 2017; Moeck et al., 2018).

## Major Approaches to Mental Control in Everyday Musical Imagery

Everyday musical imagery research can be grouped into three approaches: (1) the involuntary musical imagery approach; (2) the mental rehearsal and composition approach; and (3) the ecological musical imagery approach. Although each approach focuses on a different slice of musical imagery, all three provide support for the dual-component model.

### Involuntary Musical Imagery Approach

Recent everyday musical imagery research has focused on involuntary musical imagery—experiences defined as being “spontaneous” and “uncontrolled” (e.g., Liikkanen, 2008, 2011; Floridou et al., 2015). Although there has been some recent debate surrounding what exactly involuntary musical imagery is (see Williams, 2015 for review, and Hubbard, 2018 for a more general commentary on terminology), the “earworm”—involuntary, repetitive musical imagery—is the classic case that has received the most attention.^2^

Hearing earworms is a nearly universal experience (Liikkanen, 2008; Floridou et al., 2015), but these experiences vary. In some instances, people enjoy it (Beaman and Williams, 2010; Williamson and Jilka, 2014; Filippidi and Timmers, 2017) but in others wish the music would disappear (Liikkanen, 2011; Williamson and Jilka, 2014). Some people have a seemingly never-ending stream of involuntary musical imagery (Brown, 2006; Lipson, 2006) whereas others experience brief episodes (Floridou et al., 2015). Involuntary musical imagery can be triggered in many ways, such as by hearing a song recently (Halpern and Bartlett, 2011; Williamson et al., 2011; Hyman et al., 2013) or by personal concerns or worries (Floridou et al., 2015), or for no apparent reason (Kvavilashvili and Mandler, 2004; Hyman et al., 2013; Floridou and Müllensiefen, 2015). The common denominator is that they are involuntary.

But what exactly is meant by “involuntary” is inconsistent in the literature. In a few definitions, the involuntariness refers to initiation—the music appeared spontaneously and without intention. For example, involuntary musical imagery has been described as a “short musical piece, which comes to the mind unintended” (Floridou et al., 2017, p. 2189) and “subjectively hearing music playing in one’s mind without the individual actively retrieving it” (Filippidi and Timmers, 2017, p. 312) or as music that “intrudes into consciousness without deliberate effort” (Liikkanen, 2011, p. 237) and as “songs [that] often enter the mind without conscious volition” (Hyman et al., 2015, p. 14). Elsewhere, it appears that the focus is the inability to manage the imagery, with involuntary musical imagery being described as songs “that get stuck in your head even though you do not want them to stay there” (Beaman and Williams, 2013, p. 402) or “the experience of an inability to dislodge a song and prevent it from repeating itself in one’s head” (Beaman and Williams, 2010, p. 637). Still other definitions state that both the initiation and management are involuntary, such as a “short section of music that comes to the mind spontaneously without effort and then goes on repeating itself without conscious control” (Floridou and Müllensiefen, 2015, p. 472) or “the spontaneous recall and repetition of a piece of music within the mind” (Jakubowski et al., 2018, p. 2). The only scale of involuntary musical imagery experiences defines it as when a “short section of music comes into the mind, spontaneously, without effort, and then repeats without conscious control” (Floridou et al., 2015, p. 28).

The same term is used, but how mental control is discussed lacks consistency. Williams (2015), in his review of this literature, echoed many of these terminology concerns and concluded that involuntary musical imagery is a superordinate category that contains experiences such as earworms, musical dreams, and musical synesthesia. To Williams (2015), the key feature is that these experiences must be involuntarily initiated. More recently, researchers have also induced involuntary musical imagery in the lab (e.g., Hyman et al., 2013; Floridou et al., 2017; Moeck et al., 2018), demonstrating the ability to also examine involuntarily initiated musical imagery in the lab setting.

Although management is not explicitly referenced as a separate aspect of mental control, the involuntary musical imagery literature does provide evidence for its existence. This literature often asks about people’s ability to either change the content of their musical imagery or to end the experience. For example, in two separate studies participants were asked what activities they engaged in to get rid of their involuntary musical imagery (Beaman and Williams, 2010; Williamson et al., 2014)—people reported a variety of activities including imagining a different song, thinking of something other than the music, or engaging in an external task to end the experience (e.g., chewing gum; Beaman et al., 2015). Additionally, a small portion of people (3%) said that they never attempt to get rid of their involuntary musical imagery (Williamson et al., 2014). Other work also finds that people who find it more difficult to get rid of their involuntary musical imagery tend to have longer episodes of involuntary musical imagery and find them more worrying (Beaman and Williams, 2013). Although correlational, this suggests there are individual differences in management ability. Interestingly, findings pertaining to these types of items are not described as instances of control and sometimes episodes of involuntary musical imagery are described as malleable, involuntary experiences and that there are “many anecdotal descriptions that people successfully use active behaviors to manage their [involuntary musical imagery]” (e.g., Williamson et al., 2014, p. 2). Additionally, several involuntary musical imagery studies have examined the length of these experiences, further suggesting that this type of musical imagery can be sustained over time (e.g., Beaman and Williams, 2010; Floridou et al., 2015; Hyman et al., 2015).

One study has directly compared involuntary and voluntary musical imagery experiences using a Tempo Judgment paradigm (see Table 1; Jakubowski et al., 2018). They compared people’s perceptions of tempo for involuntarily and voluntarily initiated musical imagery of the same song and found that representation of the tempo for the two episodes was not significantly different. Like the auditory imagery studies using a similar paradigm, this suggests people can sustain involuntary musical imagery episodes. Only a few studies have assessed management, but the inclusion of these types of items suggests multiple ways in which musical imagery can be involuntary and that just because a musical imagery experience begins spontaneously, it does not mean that it always remains uncontrolled.

### Mental Rehearsal and Composition Approach

Unsurprisingly, musicians also report using imagery techniques to enhance their performances. To bolster their confidence, some performers report picturing themselves having won a role and performing on stage prior to an audition (Bowes, 2009); others rely on motor imagery to rehearse without fatigue (Bowes, 2009). Musicians use a range of mental imagery techniques, including musical imagery, as tools to improve upon their craft. Research examining musical imagery as part of the musical process—composition and rehearsal—implies that control is a key component of these uses of musical imagery. Although this work does not explicitly use control as a term, descriptions from musicians often imply they rely on controlled forms of musical imagery as a part of their craft.

#### Mental Rehearsal

Most work has focused on people’s use of mental imagery as a rehearsal tool and the efficacy of imagery compared to other rehearsal techniques. In qualitative studies, musicians described the ways musical imagery factors into their rehearsal and performance practices. Musical imagery can be used to achieve several goals—a survey of music students and musicians revealed musical imagery is often used to achieve mastery of a piece, assess technical aspects of music, and rehearse whole pieces (Gregg et al., 2008). Additionally, musicians describe using musical imagery across all stages of musical performance, from their initial learning of a piece (Holmes, 2005) to rehearsals of their repertoire (Holmes, 2005; Bowes, 2009; Fine et al., 2015) and immediately before and during their performances (Holmes, 2005; Bowes, 2009; Keller, 2012; Saintilan, 2014; Fine et al., 2015).

Musicians allude to both initiating and managing their musical imagery experiences. Most frequently, musicians reported running through the piece of music and constructing an image of how a performance should sound (Bowes, 2009; Saintilan, 2014; Fine et al., 2015). For some, there are particular situations in which they employ musical imagery (e.g., “I use [imagery] most specifically in the wings before a performance, or the hallways before an audition,” Bowes, 2009, p. 152). In other cases, musical imagery is used to achieve specific outcomes, from improving technical elements of a piece (e.g., “to locate the musical passages that have some kind of difficulty at the rhythmic level, melodic, technical and mentally seek a solution,” Bailes, 2009, p. 74; Fine et al., 2015) to meeting stylistic and artistic goals (e.g., “I will hear in my head how I want the first note to sound and the mood I want to convey,” Holmes, 2005, p. 225). The musicians do not directly say these experiences are controlled, but it is probable that to achieve these specific outcomes a portion of their musical imagery experiences are intentionally initiated (e.g., using imagery at a specified time) and managed (e.g., mentally working through technical components of a piece).

These studies examining mental rehearsal provide a limited amount of support for the use of initiation and management and any conclusions are largely speculative. These descriptions do, however, identify starting points for work emphasizing when and how musicians employ involuntary and voluntary musical imagery.

There are also quantitative studies examining similar questions that are largely concerned with the effectiveness of using musical imagery as a rehearsal technique as compared to other methods. Multiple studies manipulate the kind of feedback available to participants when they perform and assess impacts on performance quality. In one project, the efficacy of purely mental or physical practice of novel music was compared (Bernardi et al., 2013). Participants provided a baseline performance of unfamiliar piano music and then completed two sessions of either mental imagery or physical practice. The effects for physical practice were stronger, but mental practice did lead to multiple positive performance outcomes—fewer errors, better movement timing, and quicker wrist movements—suggesting purposefully initiated and maintained mental rehearsal does have benefits. Other work has manipulated the type of imagery people used—motor imagery (imagining only movement associated with performance) or non-motor musical imagery (Johnson, 2011). Like Temporal Accuracy tasks (see Table 1), participants indicated when they had mentally completed the musical excerpt. Although accuracy did not differ between the imagery conditions, using non-motor musical imagery resulted in greater confidence in tempo accuracy.

An early study assessed when using mental rehearsal is beneficial (Rubin-Rabson, 1941). While learning a new piece, musicians either (1) completed 5 physical practice trials, 4 min of mental rehearsal, and continued physical practice until completing one memorized trial; (2) physically practiced until completing one memorized trial then completed 4 min of mental rehearsal; or (3) physically practiced until completing one memorized trial and an additional 4 min of physical practice. The results indicated that the first method resulted in needing fewer physical practice trials to complete one fully memorized trial than the other methods, suggesting mental rehearsal is beneficial during initial learning of music.

In more complex paradigms, musicians perform pieces under a variety of feedback conditions. In one study, pianists were asked to perform memorized music under four different conditions: (1) normal playing conditions (baseline); (2) without auditory feedback (i.e., the volume on the keyboard was off); (3) without auditory or visual feedback (i.e., the volume on the keyboard was off and they could not see their hands); and (4) tapping a single key to the beat without auditory or visual feedback (Wöllner and Williamon, 2007). Performances in Condition 2 (no auditory feedback) had timing and dynamic profiles most like baseline performances. A similar study that manipulated the availability of motor and auditory feedback found that depriving performers of auditory, but not motor, feedback impaired performance accuracy as compared to baseline; however, people with better auditory imagery abilities were less affected (Highben and Palmer, 2004). Interestingly, other work found that having motor, but not auditory feedback, did not significantly alter performance dynamics and articulation as compared to having normal auditory and motor feedback (Bishop et al., 2013b). In all studies, participants used imagery to compensate for the absent feedback.

In a related design, participants were presented with a musical score that had a well-known melody from classical music embedded within it, but this melody could not be identified through visual inspection of the score but could using musical imagery (Brodsky et al., 2008). Participants read the score silently, with rhythm distractions (i.e., participant tapping a steady beat while the researcher taps a different rhythm) or with phonatory distractions (i.e., participant singing traditional folk song using the syllable la) and then listened to a melody that was evaluated as being the same as or different from the melody embedded in the score. Participants were able to perform the task, and accuracy was worse than the control condition for both distractor conditions—the two distractor conditions did not differ in accuracy. In a second experiment using the same paradigm, participants were asked to perform finger movements as if they were playing the piece during the score reading period, which improved accuracy in the rhythm distraction condition.

These quantitative mental rehearsal studies are comparable to those from the lab-based auditory imagery approach—they use behavioral methods to assess imagery ability in the lab—so they can be interpreted similarly. In all tasks, people are instructed to imagine specific passages of music. Their ability to do so indicates people can initiate musical imagery. Likewise, these tasks require people to imagine an extended passage, not just a single tone. This means that to complete the task, people are sustaining their image, one form of management. These principles easily transfer from the lab-based auditory imagery research, but these studies differ in their aims. By using richer musical stimuli that more closely approximate music that may be mentally rehearsed (e.g., scores, music from participant’s repertoire), these studies demonstrate the ability to control forms of complex musical imagery and that such techniques can be used to accomplish musical goals.

#### Mental Composition

A smaller literature has discussed the role of musical imagery in composition. Mental imagery has often been thought to be connected to the generation of creative ideas (see Daniels-McGhee and Davis, 1994), and musical imagery can play a role in the composition of original music. Perhaps the most striking example is Ludwig van Beethoven, who continued to compose remarkable music, such as his ninth symphony, after going deaf. Beethoven stated:

I carry my thoughts about with me for a long time, often for a very long time before writing them down. I can…be sure that…I shall not forget [a theme] even years later. I change many things, discard others, and try again and again until I am satisfied; then, in my head, I begin to elaborate the work…the underlying idea never deserts me. It rises, it grows. I hear and see the image in front of me from every angle (Hamburger, 1952, p. 194).

Examination of documentation of other eminent composers’ references to the use of musical imagery echoes the sentiments expressed by Beethoven—imagery is an important aspect of the composition process (Agnew, 1922). Beethoven’s remarks and descriptions of Schumann’s (Agnew, 1922) and Cowell’s (Cowell, 1926) composing strategies suggest that active management of musical imagery also occurs when composing. Additionally, composers report that inspiration often strikes in the form of spontaneous musical imagery (Agnew, 1922; Cowell, 1926; Bennett, 1976). Thus, it appears that while developing a composition sounds like an intentional act, there may also be involuntary, spontaneous bouts of progress.

Within the contemporary scientific literature, there has been very little discussion of musical imagery’s role in the composition process, and what does exist relies on theoretical musings, anecdotes, or interviews with a few composers (e.g., Bennett, 1976; Mountain, 2001; Bailes, 2009; Floridou, 2015). These reports, however, do align with the accounts of historical composers. Because musical imagery is free from the physical limitations of the composer (e.g., technical proficiency on an instrument; inability to play multiple parts simultaneously), imagery provides the composer a measure of flexibility (Bailes, 2009; Bailes and Bishop, 2012), and composers are free to experiment and tinker with the music to get closer to the final piece (Mountain, 2001; Bailes, 2009; Bailes and Bishop, 2012). Additionally, composers also report involuntarily initiated episodes of novel musical imagery that they may subsequently use in their own compositions (Floridou, 2015). Based on the limited evidence, there are reports describing both the initiation and management of musical imagery related to composition. But because investigations of musical imagery during the composition process are few and far between and purely descriptive, additional research with broader samples must be done to draw generalizable conclusions.

### Ecological Musical Imagery Approach

The final approach uses ecological momentary assessment techniques to measure everyday musical imagery experiences. Researchers tend to take a descriptive, exploratory approach: they seek to describe people’s everyday musical imagery experiences. Experience sampling, the most frequently used technique, collects probe-caught musical imagery experiences as they are happening via completion of multiple surveys per day across several days at random time intervals. This method provides researchers with a measure of control over data collection in people’s everyday environments (e.g., when people can complete surveys, how frequently people are probed). All studies discussed used experience sampling methods. This approach preserves differences between episodes that can be obscured when using retrospective survey or interview measures that require respondents to pool their musical imagery experiences (see Cotter and Silvia, 2017 for additional details).

This small collection of studies captures involuntary, voluntary, and creative musical imagery experiences and discuss musical imagery as a general phenomenon experienced by musicians and non-musicians alike.^3^ This research has found that musical imagery is frequent (Beaty et al., 2013; Bailes, 2015; Cotter et al., 2018), usually pleasant (Beaty et al., 2013; Cotter et al., 2018), and contain both familiar and self-generated, original music (Beaty et al., 2013; Bailes, 2015). Additionally, the subjective qualities of these experiences, such as valence or vividness, vary between episodes (Cotter et al., 2018).

Much like the other approaches, however, mental control has not been a prominent focus. Most studies in this approach have not differentiated between involuntary and voluntary instances of musical imagery, but some studies have asked questions, like the involuntary musical imagery approach, alluding to people’s ability to exert control over their musical imagery. In daily life, people do not frequently initiate musical imagery (Beaty et al., 2013; Bailes, 2015; Cotter et al., 2018)—when asked if they started an episode of musical imagery on purpose, people report doing so approximately 25% of the time (Cotter et al., 2018). Interestingly, when people are asked to initiate an episode of musical imagery in everyday life, both musicians and non-musicians report being able to do so most of the time (61%; Cotter and Silvia, 2018), and all participants were able to initiate musical imagery at least once during the study. Even though not the dominant way musical imagery begins, both musicians and non-musicians do report intentionally initiating musical imagery occasionally in their everyday life and are generally capable of initiating musical imagery when instructed to do so.

Researchers have also assessed people’s management of their musical imagery. Like the involuntary musical imagery approach, many of these items involved wanting to get rid of or alter the content of an episode. For instance, some work has asked if people wish the imagery contained different music (Bailes, 2007, 2015) or if they wanted the imagery episode to end (Bailes, 2007, 2015; Beaty et al., 2013). These items do not directly assess management, but endorsement of these statements indirectly implies management failure. Although the reporting of these responses was limited, people did not strongly endorse these items (Bailes, 2007; Beaty et al., 2013), implying that management failure is not the norm. Indeed, when people are asked whether they perceive control over their imagery, people report moderate levels of perceived control (Cotter et al., 2018).

**FIGURE 1:**
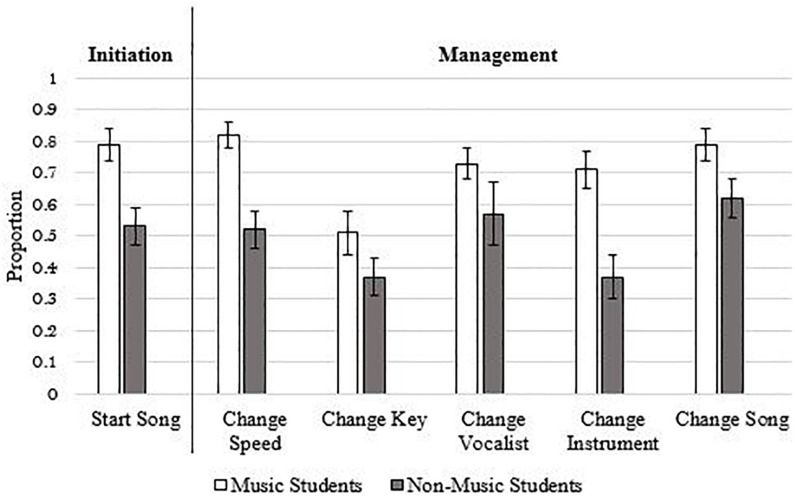
Ability to initiate and manage musical imagery. Errors bars indicate 1 SE. *N* participants = 58, *N* episodes = 1,409. Figure is adapted from Cotter and Silvia (2018).

One study has also investigated self-reported management ability (Cotter and Silvia, 2018). In this study, participants were asked to perform five manipulations to their musical imagery—changing the tempo, key, vocalist’s gender, primary instrument, and entire song. Participants reported being able to perform the various manipulations between 47 and 72% of the time (see Figure 1)—the most difficult manipulation was changing the key of the music whereas the easiest was changing the song. Unsurprisingly, people with greater musical expertise reported a greater ability to perform all manipulations. Consistent with the findings from the auditory imagery literature, people were able to manage their everyday musical imagery, but there were instances when they failed.

## Mental Control in Everyday Musical Imagery

Mental control is complex and multi-faceted. In the literature, the concept of mental control has not been explicitly developed—if directly discussed at all—and many studies treat mental control as a unitary construct. But there is a tacit understanding of its multi-faceted nature, based on how researchers describe musical imagery and measure it in practice. Thus, it is necessary to develop and recognize an explicit model of mental control in everyday musical imagery, such as the dual-component model discussed here.

### A Dual-Component Model: Initiation and Management

Of the two components, initiation—whether a musical imagery episode begins spontaneously or intentionally—has received the most interest. Because experiencing musical imagery in everyday life is common, a natural question is why we have these experiences. In the literature, this often takes the form of evaluating what triggers these experiences. In several involuntary musical imagery (Hyman et al., 2013, 2015; Floridou et al., 2015) and ecological (Bailes, 2007, 2015) studies of musical imagery, researchers have asked people why they were hearing musical imagery. Frequent responses included hearing the song recently (Hyman et al., 2013, 2015), preparing for a performance (Bailes, 2007), or not knowing exactly why (Bailes, 2007, 2015). But people do also report intentionally initiating their musical imagery. Collectively, the four literatures reviewed support initiation as one component of mental control of musical imagery.

Management, on the other hand, has not been thoroughly discussed or developed as an aspect of mental control, but it has been present in the literature. In many ways, this component is more complex than initiation because there are multiple ways in which musical imagery can be managed—altering features of the music, sustaining the experience, or stopping the episode altogether. Although these examples have not been referred to as ways to control musical imagery, several researchers assessed people’s abilities and propensities to manage their musical imagery, through evaluating different mental rehearsal techniques (e.g., Johnson, 2011) or asking people to identify manipulations to a musical excerpt (e.g., Foster and Zatorre, 2010). Overall, these literatures support management as one component of mental control of musical imagery.

But this is not the only phenomena whose underlying processes have been parsed in this manner—the initiation and management distinction presented here has its antecedents in discourse of related phenomena. In the memory literature, we can view the distinction between memories that are retrieved directly through automated activation of a memory from environmental cues and retrieved generatively through actively accessing a specific memory (e.g., Addis et al., 2012; Anderson et al., 2017) as initiation-related distinctions. Similarly, we can discuss rehearsal of memory (e.g., Craik and Watkins, 1973) as a management-related process. In the physical movement literature, research distinguishes between voluntary and involuntary actions and shows that actions that begin involuntarily can be brought under control (De Havas et al., 2016).^4^ In the more closely related visual imagery and mind wandering fields, there are also similar distinctions. There exist separate self-report measures of the ability to generate and manipulate visual images (e.g., Gordon, 1949; Sheehan, 1967; Marks, 1973; Halpern, 2015). In the mind-wandering literature, Smallwood (2013) introduced the process-occurrence model that also differentiates between how an instance of mind-wandering begins and how it unfolds. The proposed dual-component model of mental control of musical imagery is grounded in these prior conceptualizations of control. This similarity demonstrates that distinguishing between control processes is fruitful for better understanding each phenomenon and can be a guiding force for further development of the field.

## Looking Back and Looking Forward: The Utility of a Dual-Component Model

### What Can a Dual-Component Model Tell Us About Past Research?

Past research has not emphasized mental control, yet there is still valuable information about these processes in past research. With the development of this model of mental control, we can re-examine the literature with an eye toward this mental control distinction. Specifically, differentiating between initiation and management in this dual-component model will help researchers interpret and organize the large amount of prior work in seemingly disparate literatures. Past research’s assessments of initiation or management tend to be a few items given only a passing mention in articles (Beaman and Williams, 2010, 2013; Liikkanen, 2011; Williamson et al., 2014). But by re-examining the literature through the lens of the proposed model of mental control, we can better understand and interpret prior work, such as identifying relationships between the two components of mental control or recognizing limitations that were not readily apparent (see Table 2).

**TABLE 2:** Summary of implications of the dual-component model.

**How does a dual-component model shape our perspective on existing work?**
Re-examining and analyzing existing data
• Fully analyzing items related to initiation or management processes • Revising interpretations of past work through the perspective of the dual-component model
Identifying weaknesses and limitations of past work
• Lack of precision in currently used terminology (e.g., involuntary musical imagery) • Recognize limitations of work treating control as a unitary construct and identify ways to clarify existing findings through future work
Drawing parallels between approaches to musical imagery
• Identify ways in which mental control operates and is assessed similarly across different literatures and experiences • Extend analyses of similarity to other dimensions of musical imagery experiences
**How does a dual-component model shape future research?**
Combine aspects of different research approaches
• Identify complementary strengths and weaknesses of the different approaches to create new paradigms for future research • Expand upon initial research blending these approaches to examine musicological features that potentially influence mental control processes
Foundation for further refinement of the model
• Empirically evaluate candidate sub-components of management (e.g., sustention, manipulation, termination) • Further develop theory regarding cognitive mechanisms involved in initiation and management

For example, in the involuntary musical imagery approach emphasizing involuntarily initiated experiences, there are several items (e.g., whether people attempted to stop or change the experiences) that can be used to better divide and understand this class of experience. Based on existing data, it is possible to further differentiate cases of involuntary musical imagery that persist in being uncontrolled from cases in which people manage the experience. This additional specificity allows for more refined examination of whether these experiences differ on other dimensions of musical imagery. But this also suggests that current work on involuntary musical imagery is limited because it obscures potential differences between involuntarily initiated musical imagery that remains involuntary and episodes that are deliberately managed. Further, the proposed model describes multiple ways in which a single episode of musical imagery can be involuntary, showing that the label of “involuntary musical imagery” does not convey the same specificity it once did when the field of musical imagery research was less mature.

Additionally, the four literatures have evolved independently with few bridges between them. Researchers commonly review work within their own approach and provide limited, if any, consideration of findings from the other approaches. This is especially true of the mental rehearsal and composition approach, rooted in music education, which is almost completely isolated from the others. But in providing a common language regarding mental control, this model can illuminate how this literature fits alongside the other three. Although this conceptual analysis focuses on one piece of musical imagery experiences, it demonstrates these approaches have similarities.

For instance, musicians reference having music pop into their heads that is then applied to compositions (Agnew, 1922; Cowell, 1926; Hamburger, 1952) or having music they are rehearsing repeating in their minds (e.g., Holmes, 2005), suggesting that involuntary musical imagery processes—a different everyday musical imagery approach—may also play a role in the creation and rehearsal of music. But we can also identify links between lab-based auditory imagery research and musicians’ applications of imagery. Musicians report sustaining and mentally playing through music in their repertoire (e.g., Holmes, 2005; Bowes, 2009) similar to the paradigms used in several Pitch Discrimination, Timing Judgment, Tempo Accuracy, Lyric Comparison, and Loudness Profile tasks used to assess management abilities in the auditory imagery literature. Without the terminology and conceptual framework introduced here, drawing such parallels is not as straightforward.

### How Can a Dual-Component Model Guide Future Research?

Since both initiation and management are evident in the four approaches, a natural step is to apply the lessons from one approach to the others. For example, the strengths of the lab-based auditory imagery approach include its behavioral measurement and emphasis on the cognitive processes underlying imagery. But it often lacks the complexity of musical imagery in everyday life and has limited ecological validity. The ecological approach has the opposite character—research assesses musical imagery in people’s daily lives, but its descriptive and self-report nature does not provide the same clarity and validity as the lab-based studies (see Hubbard, 2013, 2018; Table 2).

Borrowing techniques would benefit both literatures without compromising their respective focuses. Future auditory imagery work could use more complex stimuli, such as music similar to what is heard in everyday life (e.g., pop songs; Bailes, 2015), to evaluate people’s control abilities. Researchers have begun using realistic stimuli in lab-based work (e.g., Godøy et al., 2006; Bishop et al., 2013a; Jakubowski et al., 2015; Weir et al., 2015) and found that people are able to generate relatively accurate musical images of the stimuli. To build upon these studies, additional work should examine how realism of stimuli and related factors (e.g., stimulus complexity, familiarity, instrumental vs. vocal) relate to mental control. Given the considerable heterogeneity of everyday musical imagery contents (e.g., Bailes, 2015; Jakubowski et al., 2016), examination of how musicological factors (e.g., presence of lyrics, complexity of composition, musical genre) influence mental control abilities is a top candidate for future research.

Conversely, future ecological studies should work to adapt the behavioral approaches used in lab-based research to increase the validity of reports. Indeed, there are a few studies that have begun to integrate behavioral and ecological assessment (e.g., singing involuntary musical imagery episodes into a recorder, McNally-Gagnon, 2016; recording tempo of voluntary and involuntary musical imagery via tapping the beat, Jakubowski et al., 2018). With the widespread use of smartphones, future research could, for example, have participants complete Pitch Discrimination or Timing Judgment tasks (see Table 1) by listening to musical excerpts and completing specific management tasks that can be used to evaluate the accuracy of their imagery. In this way, the ability to perform these tasks could be assessed across multiple environments and compared to the lab-based findings. This is only one example of how borrowing from other approaches to musical imagery can enrich the field.

Finally, the introduction of this model represents a starting point for formal investigations into the role of mental control in everyday musical imagery and allows researcher to ask many more research questions that otherwise would have remained non-obvious. How do management behaviors differ between voluntarily and involuntarily initiated imagery? Are purposefully initiated episodes easier to manage than involuntary initiated ones? Which personal or environmental factors (e.g., mood, mental fatigue) most closely relate to attempts to control musical imagery? Do these factors differ for initiation and management? These are a few examples of research that can emerge through differentiating between the initiation and management.

Importantly, future research must also investigate these components of mental control of musical imagery concurrently across all approaches to further substantiate the distinction between initiation and management. Since research has yet to examine initiation and management within the same study, the association between these two components and any differential relations with related concepts (e.g., affective valence of the experience, Cotter et al., 2018) are still an open issue. The first step would be to include measures of both initiation and management within the same study to understand the relationship between the two components. This conceptual analysis represents the starting point for such investigations and provides a theoretical basis grounded in four approaches to musical imagery and models of related phenomena (e.g., mind wandering, Smallwood, 2013).

One limitation of this model that should be addressed by future research is that sustaining, altering, and stopping musical imagery are considered examples of management processes but may instead represent sub-components of management or entirely separate components. As past work has not differentiated between ways of controlling musical imagery, the proposed model takes a more general approach through distinguishing between control of the start and evolution of an episode. But it is possible that these examples of management rely on differing, but related, cognitive processes or have different implications for people’s musical imagery. For instance, deliberately sustained episodes may have more positive valence than episodes that are altered or stopped. Research could also explore the relation between voluntarily and involuntarily managed episodes of musical imagery (e.g., Is it easier to manipulate an episode that is being voluntarily sustained than one that continues involuntarily? Are involuntarily sustained episodes more difficult to stop?). To date, no research has examined sustention, manipulation, and termination of musical imagery episodes individually and thus a parsimonious approach to mental control of musical imagery was adopted. Further work is necessary to better understand how these dimensions of management relate to one another and whether there are additional components of mental control of musical imagery.

## Concluding Remarks

People have rich internal worlds. Musical imagery is one complex, idiosyncratic internal experience the vast majority of people report regularly having in their everyday lives (e.g., Liikkanen, 2011; Cotter et al., 2018). We exhibit considerable variety in the contents of our musical imagery (e.g., Bailes, 2015), and from episode to episode our imagery differs in richness and multimodality (e.g., Bailes, 2007; Bowes, 2009). Research has examined a multitude of qualities of musical imagery, such as valence, length, and vividness (e.g., Cotter et al., 2018), but less attention has been given to the processes underlying these complex experiences.

Mental control, an important element of musical imagery experiences, is a complex, multi-faceted process that has not been a focal point of past research. Examining four diverse approaches to musical imagery—lab-based auditory imagery, involuntary musical imagery, mental rehearsal and composition, and ecological musical imagery—demonstrates that mental control is not a singular, unitary construct and can be broken down into two overarching components: initiation and management.

Recognizing a dual-component model of mental control advocates for a thoughtful re-examination of past work and generates new directions for future research. By re-examining the literature, we can identify its limitations and what prior research already can tell us about initiation and management and where there are paths for growth. But more importantly, this dual-component model can spark new lines of research to develop our understanding of the underlying processes of musical imagery.

## Author Contributions

KC is the sole author and is responsible for all manuscript contents.

## Conflict of Interest Statement

The author declares that the research was conducted in the absence of any commercial or financial relationships that could be construed as a potential conflict of interest.

## Footnotes

1 Here, I emphasize the cognitive and behavioral aspects of mental control in musical imagery; of course, such a review of imagery invites a discussion of the brain and its underlying neurobiological correlates, but the complex neuroscience of musical imagery is best reserved for a separate paper.

2 Involuntary musical imagery should not be confused with musical hallucinations, which are instances of musical imagery that are due to the presence of neurological or psychiatric disorders (Evers and Ellger, 2004; Hemming and Merrill, 2015). The literature on musical hallucinations are not reviewed here as the emphasis is on non-clinical forms of everyday musical imagery.

3 Some studies within the involuntary musical imagery approach (e.g., Beaman and Williams, 2010; Jakubowski et al., 2018) have also used ecological momentary assessment techniques; however, the ecological musical imagery approach is defined both by its methodology *and* its emphasis on musical imagery in a general sense (i.e., not emphasizing a particular type of experience), these involuntary musical imagery studies are not also reviewed in this section.

4 Although physical movement is not solely an internal cognitive process, the inhibition of thoughts can be modeled similarly to the inhibition of actions (e.g., Logan, 1983). Additionally, with the neurological connections between motor imagery and action (Berna et al., 2012) and co-occurrence of motor and musical imagery (Lotze, 2013), these distinctions provide further support for differentiating between control processes in musical imagery.
